# Controllable preparation of Ni nanoparticles for catalysis of coiled carbon fibers growth

**DOI:** 10.1186/1556-276X-9-370

**Published:** 2014-07-29

**Authors:** Xian Jian, Zuowan Zhou, Sixin Wu, Lei Chen, Qing Zeng, Chao Wang

**Affiliations:** 1 Clean Energy Materials and Engineering Center, School of Energy Science and Engineering, State Key Laboratory of Electronic Thin Films and Integrated Devices, University of Electronic Science and Technology of China, Chengdu, Sichuan 611731, China; 2 Key Laboratory of Advanced Technologies of Materials (Ministry of Education), School of Materials Science and Engineering, Southwest Jiaotong University, Chengdu, Sichuan 610031, China

**Keywords:** Carbon coil, Nickel, Nanoparticle, Catalyst

## Abstract

The mass preparation of high-purity coiled carbon fibers (CCFs) remains challenging due to the high complexity and low controllability of reaction. In this work, a controllable growth of Ni particles was fulfilled by liquid phase reduction of nickel sulfate with hydrazine hydrate. The impacts of the reaction temperature, NaOH concentration, and reaction time on the particle size and purity were investigated. The as-deposited Ni particles were characterized by scanning electron microscopy and X-ray diffraction. In addition, these Ni particles were also applied in preparing high-purity CCFs both on graphite and ceramic substrates. The diameter of the as-grown carbon microcoil was about 500 nm, and the related growth mechanism was discussed.

## Background

Coiled carbon materials exhibit a variety of unique characteristics, such as super-elasticity [1], wide band absorption of electromagnetic waves [2], and hydrogen adsorption [3]. In particular, researchers have focused on the preparation [4-9], characterization [10,11], and growth mechanism [12,13] of the coiled carbon materials because these helical materials are currently not commercially available and they possess great potential applications [14-18]. At present, artificial coiled structures at the mesoscale usually have simple helical geometries of one-dimensional helical fibers depending on the growth condition such as temperature, flow rate, and carbon source.

It was reported that several coiled carbon fibers (CCFs) can be obtained using appropriate catalyst on some substrate or with the help of electric and magnetic field. For example, Chen and Motojima prepared the carbon microcoils by the Ni-catalytic pyrolysis of acetylene containing a small amount of thiophene [19]. Three-dimensional (3D) spring-like carbon nanocoils were obtained in high purity by the catalytic pyrolysis of acetylene at 750°C to 790°C using a Fe-based catalyst, and the nanocoils have a tubular shape of diameter of about 10 to 20 nm [20]. Besides, the carbon nanocoils having coil diameters of 50 to 450 nm can be obtained by applying a magnetic field in the reaction zone or using sputtered thin films of Au and Au/Ni as catalysts [21].

In fact, Ni catalyst plays a significant role in control of the helical structure during the growth of carbon coils [1]. Though several methods of preparing nickel particles, such as hydrothermal reduction technique [22], electrodeposition [23], sol-gel process [24], and microwave irradiation method [25] have been reported, the agglomeration of the particles should be prevented or else this would result to the nonuniformity of the as-prepared Ni particles. One of the crucial factors to obtain high-purity CCFs is the controllable synthesis of catalyst nanoparticles. Since Ni grain is one of the most typical catalysts for carbon microcoil (CMC), it is necessary to synthesize uniform Ni particles with designed sizes and to study the effects on the preparation and growth mechanism of the Ni particles. In this study, we prepare Ni nanoparticles by reduction of nickel sulfate with hydrazine hydrate employing the surfactant polyvinylpyrrolidone (PVP) to prevent agglomeration of particles. The as-prepared Ni particles were also used for the growth of CCFs.

## Methods

### Materials

Nickel sulfate (NiSO_4_ · 6H_2_O, analytical reagent (AR)), PVP (K30, AR, average molecular weight 40,000), sodium hydroxide (NaOH, AR) and hydrazine hydrated (N_2_H_4_ · H_2_O, AR) were purchased from Chengdu Jinshan Chemical Reagent Limited Company, Chengdu, China. Acetylene (C_2_H_2_, 99.9%), nitrogen (N_2_, 99.999%), and hydrogen (H_2_, 99.99%) were purchased from Chengdu Liuhe Chemical Industry, Chengdu, China. All reagents were used without any further purification.

### Preparation of Ni nanoparticles

Two kinds of solution were firstly prepared. Solution A was formed by adding NaOH solution (0.8 to 1.5 M) in 20 ml hydrazine hydrated (6 M) with pH ranging from 10 to 14. Solution B was formed by dissolving 5.256 g of nickel sulfate (NiSO_4_ · 6H_2_O) in distilled water, which contained 1 g of PVP polymer as dispersant. Solution A was added to a beaker with a capacity of 100 ml and was magnetically stirred for 15 min at 60°C ~ 80°C. Then, slowly dropwise, adding solution B into A, it was stirred continuously for 45 min. The black precipitates were separated from the mother liquor by magnetic separation and washed repeatedly with distilled water and acetone until the pH was 7. The grey-black powder was finally dried in vacuum at 25°C.

### Preparation of coiled carbon fibers

The as-prepared Ni nanoparticles were used as catalyst for CCFs and dispersed on a graphite substrate by spraying and drying the suspension of Ni particles. Then CCFs were obtained on the graphite by catalytic pyrolysis of acetylene containing a small amount of thiophene as the liquid catalytic addictives. Acetylene, hydrogen, and nitrogen were introduced into a horizontal reaction tube (quartz, 28 mm i.d.) which was heated from the outside by a tubular furnace. The flow rates of acetylene and nitrogen were fixed at 20 and 60 ml/min (sccm), respectively, and the hydrogen flow rate ranged from 100 to 140 sccm. Several kinds of CCFs grew exclusively on the upper region of the source gas steam.

### Characterization

The crystal structure of catalyst particles and helical carbon fibers was investigated using X-ray diffraction (XRD with Ni filter, Panalytical X’Pert PRO diffractometer, Almelo, the Netherlands). The size and morphology analyses of nickel particles and CCFs were performed using environmental scanning electron microscopy (ESEM; FEI, Quanta 200, FEI Company, Hillsboro, OR, USA) with an accelerating voltage of 20.0 kV and high-resolution transmission electron microscopy (HRTEM; JEM-2100 F, JEOL Ltd., Tokyo, Japan) at an accelerating voltage of 200 kV.

## Results and discussion

### Effects on the preparation of Ni particles

To obtain controllable catalyst particles, factors, such as reaction temperature and time, pH values, and the concentration of nickel ions, should be considered. Among these factors, reaction temperature and pH value were addressed in the following discussion.

#### Effect of reaction temperature

**Figure 1:**
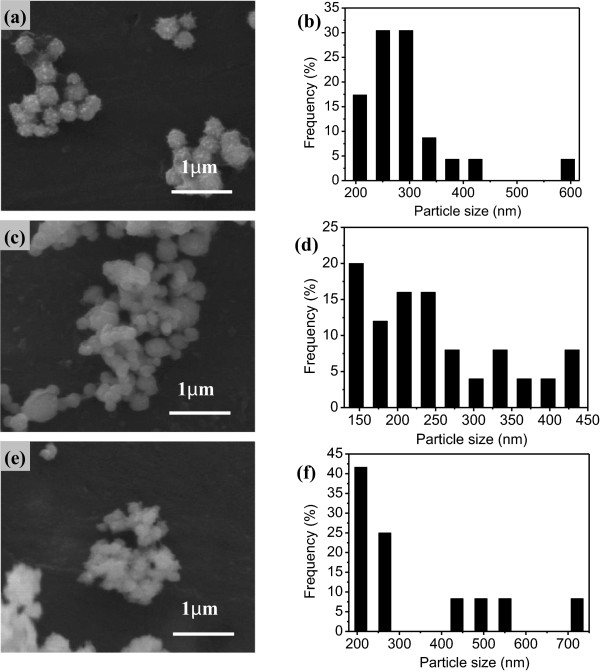
**SEM images and size distributions of nickel particles at different temperatures.** SEM images **(a,b,c)** and size distributions **(d,e,f)** of nickel particles obtained with different reaction temperatures: **(a,b)** 60°C, **(c,d)** 70°C, and **(e,f)** 80°C.

The effect of reaction temperature on the preparation of nickel powders was experimentally investigated when the NaOH solution was 1 M (mol/l). The chemical reduction was performed at various temperatures including 60°C, 70°C, and 80°C. Figure 1a,c,e shows the scanning electron micrographs of the samples obtained at designed temperatures. From the scanning electron microscopy (SEM) results, the particles in all of samples are spherical in shape and agglomerated sometimes. In the sample prepared at 60°C, the particle size distribution is broad and the surface is rough. The spherical nickel particles contain a number of ultra small particles of less than 50 nm in diameter. While for the samples prepared at higher temperature, say 70°C, the particle size distribution is relatively narrow and the surface turns smooth. When the reaction temperature reaches 80°C, the particles become cottony and the particle size distribution seems broad again. The particle size distributions for each sample were determined by software Nano Measurer 1.2.5 using enlarged SEM images as shown in Figure 1b,d,f. The average particle sizes of powders obtained at 60°C, 70°C, and 80°C are 294.6, 247.6, and 333.2 nm, respectively. From the analysis of particle size distribution, the average diameter of the particles at 70°C has the relatively smaller particle size with a wide distribution of 133 to 440 nm. This phenomenon indicates that the average particle size is strongly affected by the reaction temperature. Separation of the nucleation and the growth are the premise of the formation of controllable particles. We suppose that homogeneous nucleation occurs until a nucleus of critical size is obtained at critical reaction temperature, such as about 70°C in this case.

#### Effect of NaOH concentration

**Figure 2:**
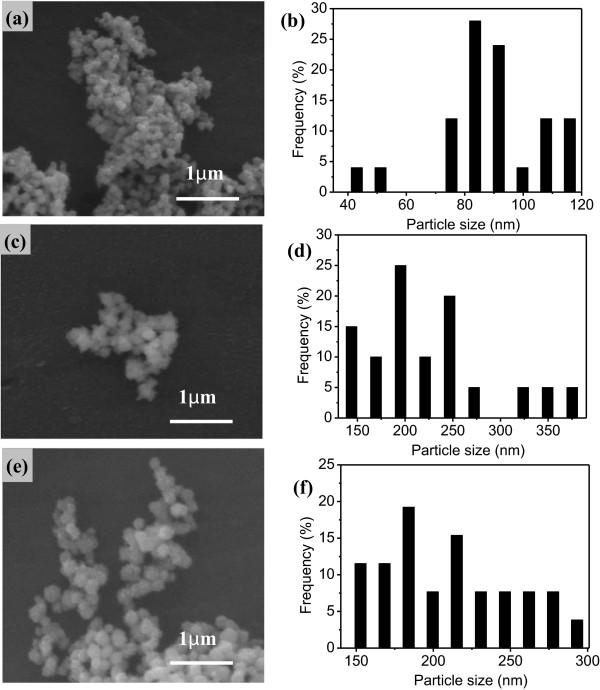
**SEM images and size distributions of nickel particles at different NaOH concentrations.** SEM images **(a,b,c)** and size distributions **(d,e,f)** of nickel particles obtained with different NaOH concentration: **(a,b)** 0.8 M, **(c,d)** 1.2 M, and **(e,f)** 1.5 M.

**Figure 3:**
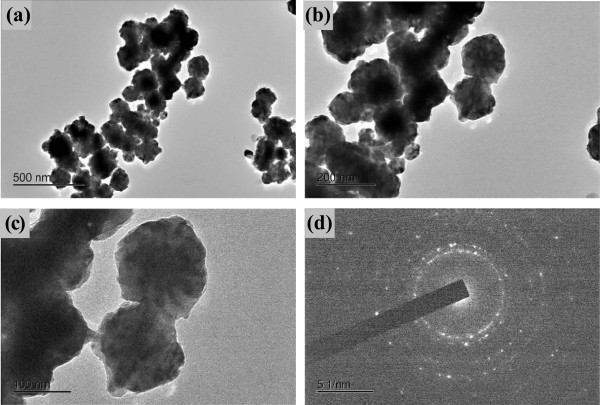
**TEM images and electron diffraction pattern of Ni nanoparticles.** TEM images **(a,b,c)** and electron diffraction pattern **(d)** of Ni nanoparticles obtained at 70°C when the molar concentration of NaOH is 0.8 M.

The effects of NaOH concentration are also investigated in the range of molarity from 0.8 to 1.5 M at 70°C. The molar concentration of NaOH solution is crucial to adjust the reaction rate. Figure 2 shows micrographs of the samples obtained at different concentrations of NaOH. The as-prepared particles are spherical in shape and without agglomeration when molar concentration of NaOH solution is 0.8 M (mol/l) as shown in Figure 2a. The average particle size was 88.3 nm with a relatively narrow distribution of 39.1 ~ 119.4 nm as denoted in Figure 2b. As the molar concentration of NaOH solution increased to 1.2 M, the obtained particle size was 224.7 nm with a wide distribution ranging from 131.7 to 387.9 nm (Figure 2d). Similarly, when the molar concentration of NaOH solution increased to 1.5 M, the average diameter became 211.1 nm (Figure 2f) with a wide distribution of 145.0 to 300.5 nm. The surfaces in the case of panels Figure 2a,c were rough. The effect of the molar concentration of NaOH solution on the size of nickel particles is discussed in terms of nickel growth mechanism.From the transmission electron microscope (TEM) observation, the as-obtained nickel particles are spherical and relatively uniform in the low-magnification TEM images in Figure 3a,b. Actually, these quasi-spherical particles contain a number of ultra small particles of less than 50 nm, as shown in Figure 3c, indicating they are Ni multicrystal which is confirmed by the electron diffraction pattern in Figure 3d.

During the formation of Ni particle, the reactions may take place as follows:

(1) $${\mathrm{NiSO}}_{4}+2\mathrm{NaOH}\to \mathrm{Ni}{\left(\mathrm{OH}\right)}_{2}↓+{\mathrm{Na}}_{2}{\mathrm{SO}}_{4}$$

(2) $$2\mathrm{Ni}{\left(\mathrm{OH}\right)}_{2}+N_{2}H_{4}\to 2N_{i}↓+N_{2}↑+4H_{2}O$$

When the molar concentration of NaOH in the NiSO_4_ solution is low, the reduction rate of nickel ion becomes slow and numerous light green clusters of Ni(OH)_2_ generate in the initial stage of reaction of about 15 min. Then Ni nanoparticles form gradually by the reduction of uniform clusters of Ni(OH)_2_ during the following 100 min. In contrast, the clusters of Ni(OH)_2_ become larger and the amount of the clusters decreases when the molar concentration of NaOH is higher than 1 M.

### Structural characterization of Ni particles

**Figure 4:**
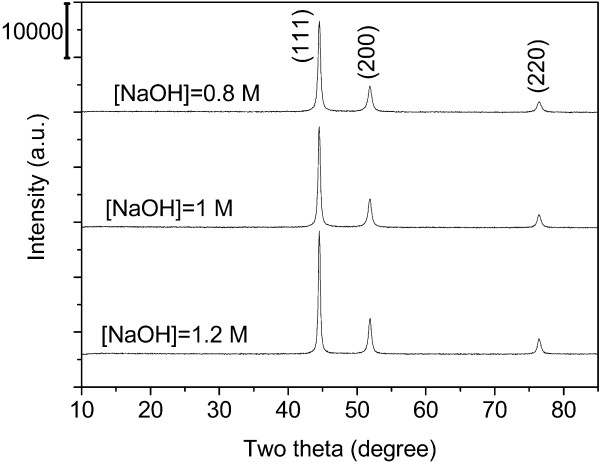
XRD patterns of nickel powder at different molar concentrations of NaOH.

The formation of nickel particles is confirmed by XRD studies. In the XRD profile (Figure 4), the three characteristic diffraction peaks of metallic copper over 40° are observed, which agrees well with the standard nickel diffraction pattern (ICDD, PDF file No. 01-070-1849). These correspond to the (111), (200), and (220) diffraction planes of only cubic Ni phase. The crystallite size of Ni for the most intense peak (111) plane was determined from the X-ray diffraction data using the Debye-Scherrer formula:

(3) $$D=\frac{\mathrm{k\lambda}}{\beta \cos\theta }$$

where *D* is the crystallite size, *k* = 0.89 is a correction factor to account for particle shapes, *β* is the full width at half maximum (FWHM) of the most intense diffraction peak (111) plane, *λ* = 1.5406 Å is the wavelength of Cu target, and *θ* is the Bragg angle. The average crystallite sizes of the produced nickel powders are reckoned as 5.07, 4.56, and 5.70 nm when the molar concentration of NaOH is 0.8, 1.0, and 1.2 M (mol/l), respectively. It is pointed that the particle sizes calculated from the XRD pattern are considerably smaller than those determined from the SEM images. The analysis suggests that the spherical nickel particles may contain a number of ultra small crystals, which agrees with the observation of morphology.

### Preparation of coiled carbon fibers and corresponding mechanism

**Figure 5:**
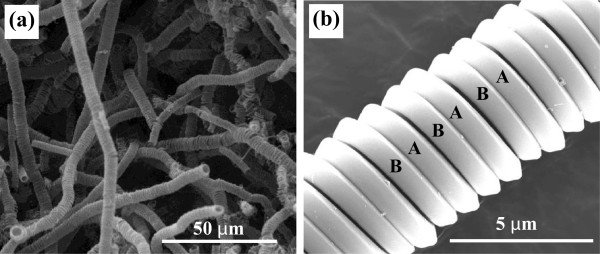
**SEM images of regular CMC.** SEM images of **(a)** low magnification and **(b)** high magnification. The regular CMC was obtained using Ni particles on graphite substrate under the following conditions: reaction temperature of 750°C, N_2_ at 100 ml/min, H_2_ at 60 ml/min, C_2_H_2_ at 20 ml/min, and bathing temperature of thiophene at 40°C. The regular CMC are made up of double helical fibers A and B.

**Figure 6:**
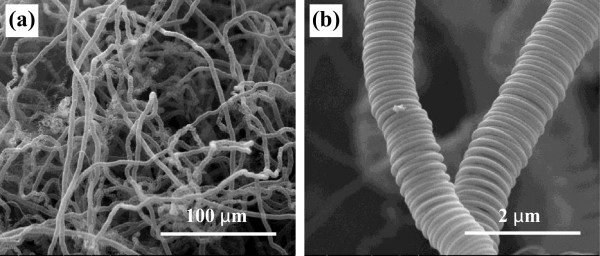
**SEM images of irregular CMC.** SEM images of **(a)** low magnification and **(b)** high magnification. The irregular CMC was obtained using Ni particles on ceramic substrate under the following conditions: reaction temperature of 750°C, N_2_ at 100 ml/min, H_2_ at 60 ml/min, C_2_H_2_ at 20 ml/min, and bathing temperature of thiophene at 40°C.

The CCFs with a constant coil diameter and coil pitch throughout a piece of the carbon coils could be obtained under the following reaction conditions: temperature of 750°C, time of 2 h, acetylene flow rate at 40 ml/min, hydrogen flow rate at 60 ml/min, and nitrogen flow rate at 100 ml/min. Meanwhile, the liquid thiophene was heated to 40°C using a water bath kettle. The catalytic addictive was introduced by the acetylene flow into liquid thiophene. From previous study [4-9], the characteristic parameters of helical carbon such as fiber diameter depend on the catalyst properties and reaction condition. To prepare high-purity carbon coils, the Ni nanoparticles prepared at 70°C, keeping the molar concentration of NaOH solution at 0.8 M, were used as catalyst for CCFs. Figure 5 displays the typical product prepared at 750°C. There are almost all carbon microcoils with regular morphology, and the CCFs are all of double helix, having an average fiber diameter of about 600 nm and coil diameter of 3 μm. Coil gap ranges from zero to several hundred nanometers. It should be noted that the nickel particle size is thinner than those of carbon fiber synthesized in this work.In further experiments, a ceramic plate was placed into the reaction tube instead of graphite substrate, and Ni catalyst was evenly dispersed in the ceramic substrate. Although other reaction conditions were unchanged, the uniformity of the as-prepared microhelix carbon fibers changes greatly as shown in Figure 6. The distortion of the helical fiber occurred randomly, indicating that the interaction between catalyst and ceramic substrate differs from graphite substrate.

**Figure 7:**
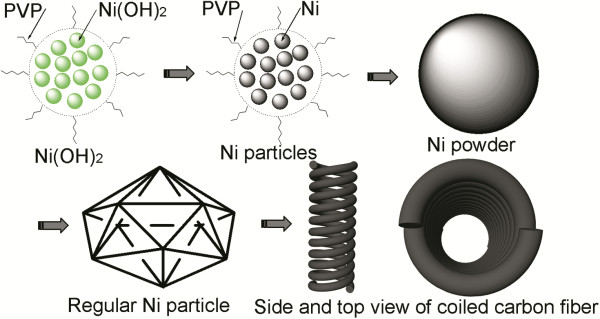
Scheme of corresponding mechanisms of nickel formation and growth of coiled carbon fiber.

It is known that the size of carbon fiber mainly depends on the size of the catalyst particle. Chen et al*.*[26] reported that carbon nanocoils with twisting form were grown by the Ni/Al_2_O_3_-catalyzed pyrolysis of acetylene. Ni particles supported on fine Al_2_O_3_ powders were prepared by an impregnation method using Ni(NO_3_)_2_ as a precursor and was used as the catalyst in their research. It is obvious that the Ni fine particles disperse well during the growth of carbon fiber due to Ni-supporter interaction in Ni/Al_2_O_3_. Though Ni catalyst nanoparticle of about 90 nm can be obtained by the induction of Ni(OH)_2_ clusters insulated by PVP, those Ni nanoparticles tend to aggregate and grow into larger Ni powder of about 600 nm because of their high surface energy and temperature action. Once the relatively large Ni powder forms, it develops gradually into regular Ni powder with catalytic anisotropy, and double helical carbon fiber begins to grow on catalyst particle. The corresponding mechanism is well visualized in Figure 7. The above analysis suggests that the parameters of carbon coil, such as fiber diameter, coil pitch and gap, are in control using suitable Ni particle.

## Conclusions

By controlling the reaction temperature and NaOH concentration, Ni nanoparticles with designed size can be obtained by reduction of nickel sulfate with hydrazine hydrate employing the surfactant of PVP. Ni nanoparticles of about 90 nm were obtained at 70°C when the molar concentration of NaOH solution was 0.8 M. The as-prepared Ni nanoparticles of about 90 nm contain some ultra small crystals less than 50 nm, and they are effective for catalytic growth of CCFs. The diameter of coiled carbon fibers is remarkably larger than that of the Ni particle catalysts. It was proposed that the aggregation and shape changes occurred during the growth of coiled carbon fiber, and the morphology of carbon helix can be adjusted by choosing the proper substrate of Ni catalyst.

## Competing interests

The authors declare that they have no competing interests.

## Authors' contributions

XJ performed all the experimental measurements and wrote the manuscript. ZZ put the basis of the entire project and make corrections to the manuscript. CW guided the internal collaboration, and read and improved the manuscript. SW, LC, and QZ did some supplementary experiments. All authors read and approved the final manuscript.
